# The MFS transporter BcTpo1 governs the oxidative stress response and infection of Botrytis cinerea

**DOI:** 10.1007/s44297-026-00074-7

**Published:** 2026-04-22

**Authors:** Lingchao Wang, Youmei Xie, Jiaxuan Li, Fugen Yang, Wenxing Liang, Qianqian Yang

**Affiliations:** Shandong Provincial Key Laboratory of Microbial Resource Exploration and Innovative Utilization, Engineering Research Center for Precision Pest Management for Fruits and Vegetables of Qingdao, College of Plant Health and Medicine, Shandong Engineering Research Center for Environment-Friendly Agricultural Pest Management, Qingdao Agricultural University, Qingdao, China

**Keywords:** *Botrytis cinerea*, Reactive oxygen species sensitivity, Spermine, Virulence

## Abstract

*Botrytis cinerea* is a devastating necrotrophic pathogen causing gray mold disease. Understanding its pathogenic mechanisms is critical for developing novel control strategies. In this study, we investigated the role of spermine and its transporter BcTpo1 in fungal virulence. Spermine, but not spermidine, specifically inhibited conidial germination, infection structure formation, and pathogenicity. Deletion of *BcTpo1* resulted in pleiotropic defects, including altered mycelial growth, conidiation, sclerotia formation, and melanin biosynthesis. Under oxidative stress, Δ*BcTpo1* exhibited reduced intracellular spermine levels compared to the wild-type strain, correlating with elevated hydrogen peroxide sensitivity and impaired infection cushion formation. Exogenous spermine partially rescued these defects by enhancing antioxidant enzyme (superoxide dismutase and peroxidase) activities. Together, these findings establish BcTpo1 as a key regulator linking spermine homeostasis, oxidative stress adaptation, and pathogenicity.

**Supplementary Information:**

The online version contains supplementary material available at 10.1007/s44297-026-00074-7.

## Introduction

*Botrytis cinerea* (*B. cinerea*), the causative agent of gray mold, is one of the most significant diseases impacting vegetable and fruit production. It has the potential to infect over 1,400 plant species, causing substantial economic losses [1, 2]. To date, chemical control remains the most effective management strategy. However, *B. cinerea* poses a high risk of developing resistance to fungicides, which threatens effective disease control and agricultural productivity [3]. Therefore, further investigation into the pathogenic mechanisms is urgently needed to identify novel fungicide targets and formulate sustainable control strategies.

Pathogen attacks trigger multilayer immune systems in host plants. The oxidative burst involves the production of reactive oxygen species (ROS), such as superoxide (O₂^•^⁻) and hydrogen peroxide (H₂O₂), which represent one of the earliest responses of plants to pathogen infection [4]. H₂O₂ can induce oxidative damage and potentially lead to cell death, thereby obstructing pathogen colonization. Additionally, H₂O₂ functions as a key signaling molecule that activates the expression of defense-related genes [5, 6]. In response, pathogens must overcome the oxidative burst by either inhibiting ROS production [7] or detoxifying the ROS generated by the host [8]. In addition to ROS, polyamines (PAs) also play a crucial role in modulating plant defense responses to phytopathogens [9–12]. Polyamines, which are small aliphatic polycations ubiquitous in eukaryotes, serve diverse functions in fundamental physiological and developmental processes, including cell division, embryogenesis, and senescence [13–15]. In plants, the major PAs are putrescine (Put), spermidine (Spd), and spermine (Spm). These compounds accumulate in plants during both abiotic and biotic stresses, with Spm predicted to act as a signaling molecule involved in plant defense responses and senescence [16, 17]. Furthermore, PA metabolism is linked to several other metabolic pathways, including ROS production [18]. Thus, PAs and H₂O₂ function as significant signaling agents in plants subjected to both biotic and abiotic stress conditions. Crosstalk between polyamine transduction and oxidative signaling has been reported [19, 20]. However, the specific interaction patterns between these signaling pathways remain unclear, necessitating further investigation.

Both pathogens and plants have the capability to produce PAs, which makes understanding their origins during interactions complex. Given that PAs can be toxic at elevated levels, their intracellular concentrations are meticulously regulated through mechanisms that control biosynthesis, degradation, and transport either to intracellular storage sites or out of the cell [21, 22]. In *Saccharomyces cerevisiae*, four transporters, Tpo1p to Tpo4p, have been identified to provide resistance to PAs [21, 22]. Tpo2p and Tpo3p specifically transport spermine, while Tpo1p and Tpo4p transport putrescine, spermidine, and spermine [21, 22]. Tpo1 belongs to the major facilitator superfamily (MFS), which has been demonstrated to play a key role in drug resistance. Moreover, Tpo1 is implicated in the adaptation of yeast to oxidative stress, indicating its additional role [23]. Consequently, Tpo1 might be integral to the tolerance of ROS tolerance and virulence of fungal pathogens. Nonetheless, the precise role of Tpo1 in fungi that are pathogenic to plants remains to be elucidated.

In this study, we aimed to elucidate the role of spermine in the pathogenicity of *B. cinerea* and to investigate whether the polyamine transporter BcTpo1 influences fungal virulence by modulating spermine homeostasis, thereby affecting ROS balance and the oxidative stress response. Our findings reveal a previously unrecognized link between polyamine transport, redox homeostasis, and pathogenicity, providing new insights into the adaptive mechanisms of necrotrophic fungi in response to host oxidative bursts.

## Results

### Spermine interferes with conidial germination, virulence and H_2_O_2_ response of B. cinerea

**Fig. 1:**
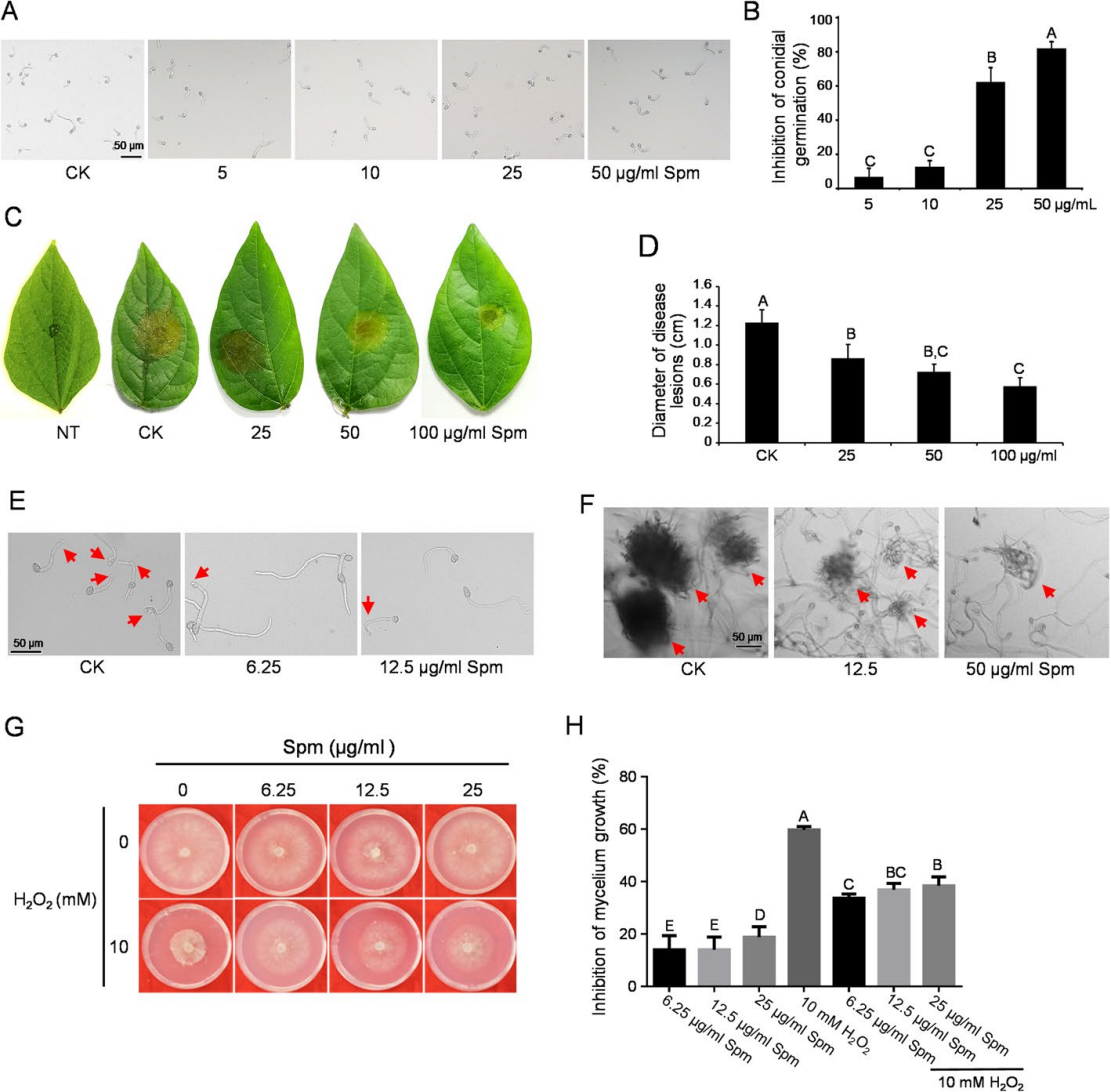
Spermine (Spm) inhibits conidial germination, virulence, and H_2_O_2_ sensitivity of *B. cinerea.* **A** Microscopic observation of conidial germination treated with or without Spm in vitro. **B** Quantification of conidia germination rates from (A). **C** Symptoms on mung bean leaves inoculated with *B. cinerea* conidia (10^5^ conidia/mL) supplemented with the indicated concentrations of Spm. **D** Lesion diameters on mung bean leaves from (C) measured at 3 days post-inoculation (dpi). **E** Spm suppresses appressorium formation in *B. cinerea*. Conidia (10.^5^ conidia/mL in 10 mM fructose) were incubated on glass slides at 25 °C for 8 h. Appressoria are indicated by red arrows. **F** Spm suppresses infection cushion formation in *B. cinerea*. Mycelium plugs were placed on slides with Spm and incubated at 25 °C for 24 h. Infection cushions are indicated with red arrows. **G** Spm reduces the H_2_O_2_ sensitivity of *B. cinerea*. **H** Inhibition of mycelial growth in *B. cinerea* following treatment with H₂O₂ and Spm. Different letters above the mean values of three replicates indicate a statistically significant difference between the samples (p < 0.05, ANOVA)

PAs are reported to be essential for cell growth; however, their specific roles in pathogenic fungi remain unclear. In this study, the effect of polyamines (Spm) and Spd) on the mycelial growth of *B. cinerea* was evaluated. In contrast to the inhibitory effects of Spm, Spd showed no significant impact on mycelial growth or conidial germination (Fig. S1), indicating a functional specificity among polyamines in *B. cinerea*. Consistent with these findings, Spm markedly reduces the virulence of *B. cinerea* (Fig. 1C, D), whereas Spd exhibits no inhibitory effect on virulence (data not shown). To further explore the mechanism underlying the control of *B. cinerea* virulence, we analyzed the formation of infection structures. As shown in Fig. 1E and 1 F, Spm significantly suppresses the formation of appressoria and infection cushions in *B. cinerea*. Thus, Spm interferes with conidial germination and the formation of infection structures, thereby reducing the virulence of *B. cinerea*.

Furthermore, PAs play a complex and dual role in regulating cellular ROS sensitivity [23]. To further investigate the role of Spm in ROS sensitivity in *B. cinerea*, we conducted phenotypic assays. The results indicated that Spm significantly reduced the sensitivity of *B. cinerea* to H_2_O_2_, suggesting that Spm can mitigate oxidative stress in *B. cinerea* (Fig. 1G, H). Collectively, these findings demonstrate that Spm can effectively reduce H_2_O_2_ levels in *B. cinerea*.

### The MFS transporter BcTpo1 is involved in mycelial growth, conidial formation, and melanin production in B. cinerea

**Fig. 2:**
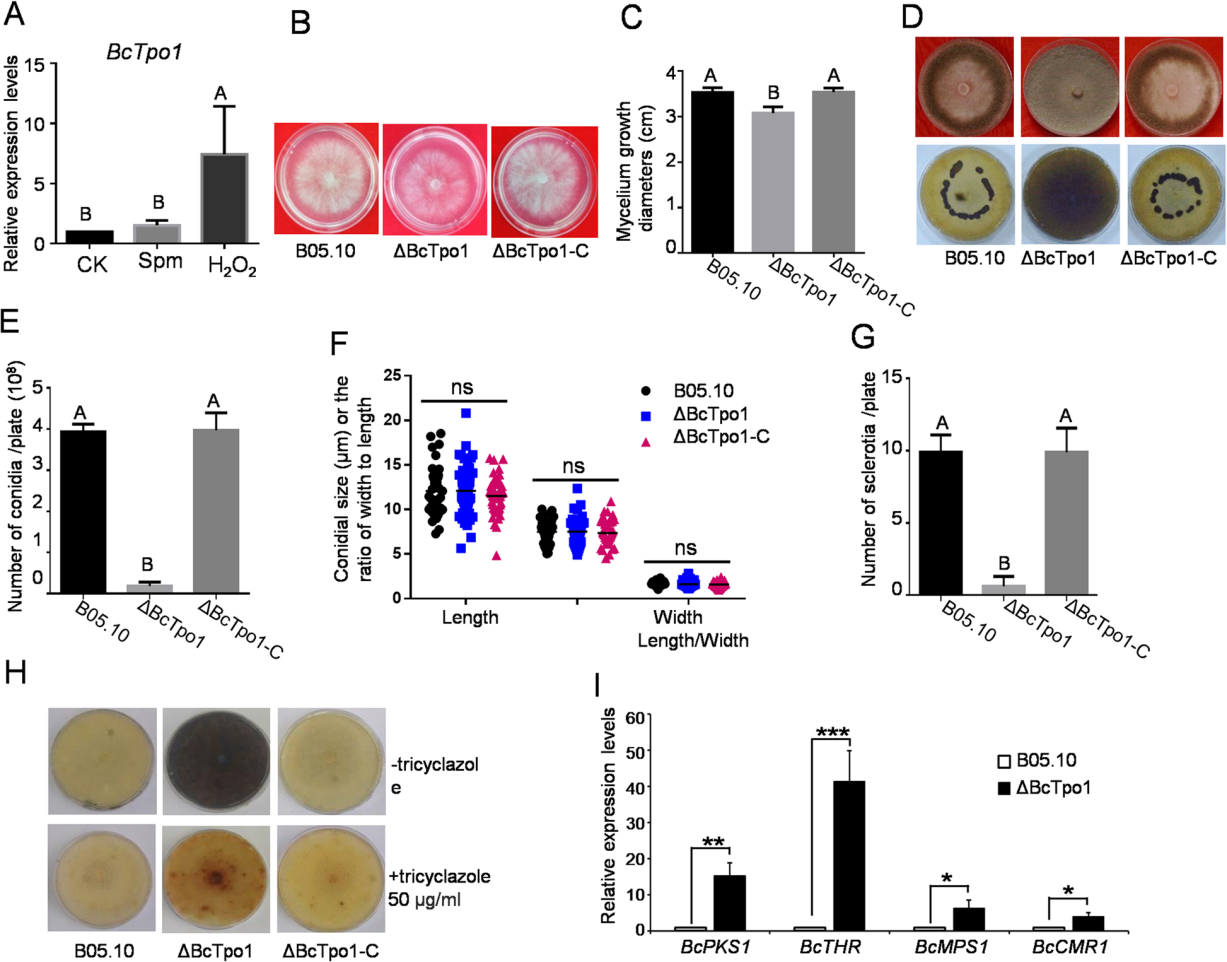
Biological functions of BcTpo1 in *B. cinerea*. **A** The expression levels of *BcTpo1* in response to Spm and H_2_O_2_. Different letters above the mean values of three replicates indicate a statistically significant difference between the samples (p < 0.05, ANOVA). **B** Colony morphology of the wild-type (B05.10), ΔBcTpo1, and complementation (ΔBcTpo1-C) strains after 60 h of incubation on PDA plates. **C** The colony diameter of the indicated strains after 60 h of incubation on PDA plates was measured. **D** Colony morphology of B05.10 and the indicated *BcTpo1* mutant strains after 10 days and 4 weeks of incubation on PDA plates. **E** The number of conidia in B05.10 and mutants. The plates were incubated at 25 °C for 10 days before the conidia were washed and counted under a microscope. **F** Conidial dimensions (length, width, and the ratio of width to length) of the indicated strains. A total of 50 conidia were measured. **G** Sclerotia production of the indicated strains. The plates were incubated at 10 °C for 4 weeks. **H** Mycelial pigmentation of the indicated strains after 9 days of growth on PDA supplemented with or without tricyclazole, a melanin biosynthesis inhibitor. **I** The relative expression levels of four melanin biosynthesis-related genes: *BcPKS1*, *BcTHR*, *BcMPS1* and *BcCMR1.* Star represents significant differences according to t test (ns, not significant; * p < 0.05; ** p < 0.01; *** p < 0.001; **** p < 0.0001)

Given that PAs play significant roles in the morphology and virulence of *B. cinerea*, it is plausible that the polyamine transporter Tpo1 is also implicated in these processes. To test this hypothesis, we evaluated the expression level of *BcTpo1* under oxidative stress and observed that it was up-regulated following H_2_O_2_ treatment (Fig. 2A), further supporting its role in the oxidative stress response. We constructed a gene deletion mutant (ΔBcTpo1) and a complementary strain (BcTpo1-C) to further investigate this hypothesis. As illustrated in Fig. 2B and 2 C, the deletion of *BcTpo1* resulted in retarded mycelial growth of *B. cinerea*, while the complementary strain completely restored this defect. Additionally, ΔBcTpo1 produced fewer aerial hyphae and exhibited a significant reduction in conidiation and sclerotia formation compared to both the wild-type strain (B05.10) and complementary strains after 8 days of culture on Potato Dextrose Agar (PDA) medium (Fig. 2E, G). Meanwhile, the conidial morphology analysis revealed no significant difference in the length or width between ΔBcTpo1 and the B05.10 or ΔBcTpo1-C strains (Fig. 2F). These results indicate that BcTpo1 plays a crucial role in mycelial growth, conidiation, and sclerotia formation in *B. cinerea*.

Moreover, ΔBcTpo1 exhibited darker pigmentation after 10 days of incubation (Fig. 2H). To determine whether this pigment was melanin, we supplemented the culture medium with tricyclazole, a known inhibitor of melanin biosynthesis [24]. Pigment production in ΔBcTpo1 was significantly diminished under tricyclazole inhibition, confirming that the pigment was indeed melanin (Fig. 2H). To further investigate the underlying mechanisms of increased melanin synthesis, we analyzed the expression of melanin biosynthesis-related genes in the mutant. The results indicated that the expression levels of several key genes, including *BcPKS1*, *BcTHR*, *BcMPS1*, and *BcCMR1*, were significantly elevated in ΔBcTpo1 compared to the wild-type strain (Fig. 2I), suggesting that the upregulation of these genes may directly contribute to enhanced melanin production.

### BcTpo1 is involved in the H2O2 sensitivity of B. cinerea

**Fig. 3:**
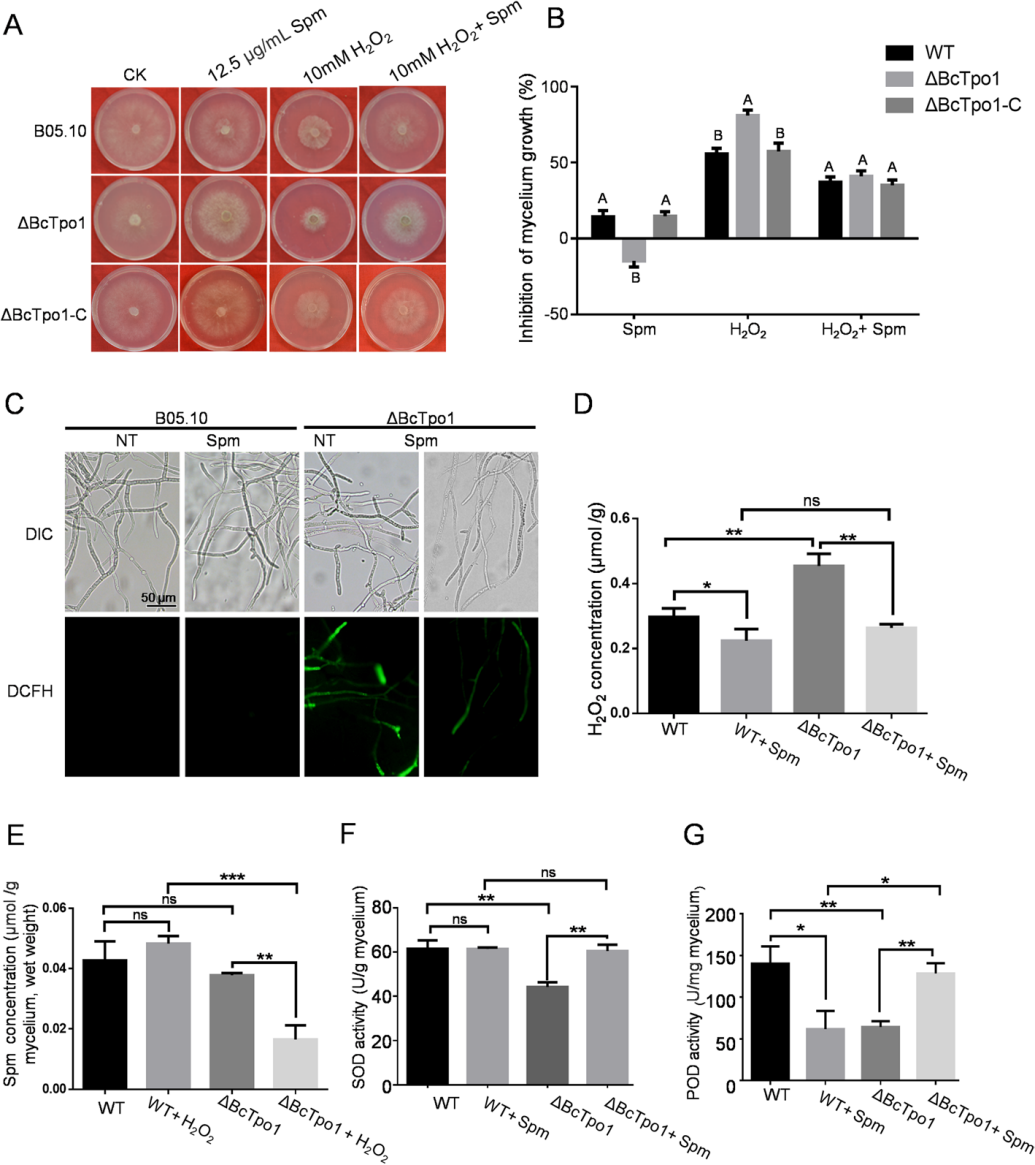
BcTpo1 is involved in the H_2_O_2_ sensitivity of *B. cinerea.* **A** Sensitivity of B05.10, ΔBcTpo1 and ΔBcTpo1-C to Spm and H_2_O_2_. Photographs were taken 60 h after incubation. **B** The inhibition growth rate of B05.10, ΔBcTpo1 and ΔBcTpo1-C to Spm and H_2_O_2_. Different letters above the mean values of three replicates indicate a statistically significant difference between the samples (p < 0.05, ANOVA). **C** ROS accumulation detected by DCFH-DA staining in mycelia of B05.10 and ΔBcTpo1 mycelia with or without Spm treatment (12.5 μg/mL for 3 h). **D** Quantification of intracellular H₂O₂ levels in B05.10 and ΔBcTpo1 mycelia under the indicated conditions. **E** The intracellular Spm concentrations in B05.10 and ΔBcTpo1 cells treated/untreated with H_2_O_2_. **F** SOD (EC 1.15.1.1) activities in B05.10 and ΔBcTpo1 mycelium in Spm-treated and untreated mycelium. **G** POD (EC1.11.1.7) activities in B05.10 and ΔBcTpo1 mycelia in Spm-treated and untreated mycelia. Star represents significant differences according to t test (ns, not significant; * p < 0.05; ** p < 0.01; *** p < 0.001; **** p < 0.0001)

Given that Spm is associated with the H_2_O_2_ response of *B. cinerea*, we investigated the relevant phenotypes of ΔBcTpo1. Notably, Spm was able to partially rescue the growth defect observed in ΔBcTpo1. The addition of exogenous Spm promoted the growth of aerial hyphae and increased the colony diameter of ΔBcTpo1 (Fig. 3A, B). Furthermore, ΔBcTpo1 displayed increased sensitivity to H_2_O_2_, with Spm mitigating this sensitivity similar to B05.10 (Fig. 3A, B). To determine whether the altered sensitivity to oxidative stress was linked to H_2_O_2_ and Spm content in the mycelium, we quantified H_2_O_2_ and Spm levels in both the wild-type and mutant strains. The intracellular ROS levels in the mutants were assessed using 2,7-dichlorodihydrofluorescein diacetate (DCHF-DA). As shown in Fig. 3C, the mycelium of ΔBcTpo1 exhibited a significantly higher fluorescence intensity compared to B05.10, indicating that BcTpo1 is associated with ROS accumulation in *B. cinerea*. Furthermore, the exogenous supplementation of Spm was found to reduce ROS levels in ΔBcTpo1 (Fig. 3C). Similar results were obtained through the measurement of intracellular H_2_O_2_ levels (Fig. 3D). These findings demonstrate that Spm not only promotes the growth of ΔBcTpo1 but also reduces H_2_O_2_ accumulation in ΔBcTpo1. Interestingly, under oxidative stress, ΔBcTpo1 accumulated significantly less intracellular Spm compared to the wild-type strain (Fig. 3E), whereas no difference was observed under normal conditions. This observation suggests that BcTpo1 may be involved in maintaining Spm homeostasis specifically during stress conditions. These findings indicate that BcTpo1 plays a critical role in ROS sensitivity and Spm levels in the fungus.

Based on these findings, we hypothesize that intracellular Spm levels may regulate the activity of antioxidant enzymes. To test this hypothesis, we further evaluated the activities of antioxidant enzymes in the mutant. As illustrated in Fig. 3F-G, the enzyme activities of superoxide dismutase (SOD), and peroxidase (POD) in ΔBcTpo1 were significantly decreased compared to B05.10. Meanwhile, SOD and POD activities were significantly increased in the presence of exogenous Spm compared to the control of ΔBcTpo1 (Fig. 3F-G).

Furthermore, our supplemental qRT-PCR analysis revealed that in the wild-type strain, exogenous Spm treatment led to downregulation of antioxidant enzyme-encoding genes (SOD and CAT, etc.) and their regulators (BcAp1 and BcSkn7) (Fig. S2). In contrast, in ΔBcTpo1, these genes were already upregulated under basal conditions due to elevated ROS levels, and exogenous Spm further enhanced their expression. These results indicated that the regulation of SOD and POD activities by exogenous Spm in ΔBcTpo1 likely involves indirect effects.

### BcTpo1 is essential for the virulence of B. cinerea

**Fig. 4:**
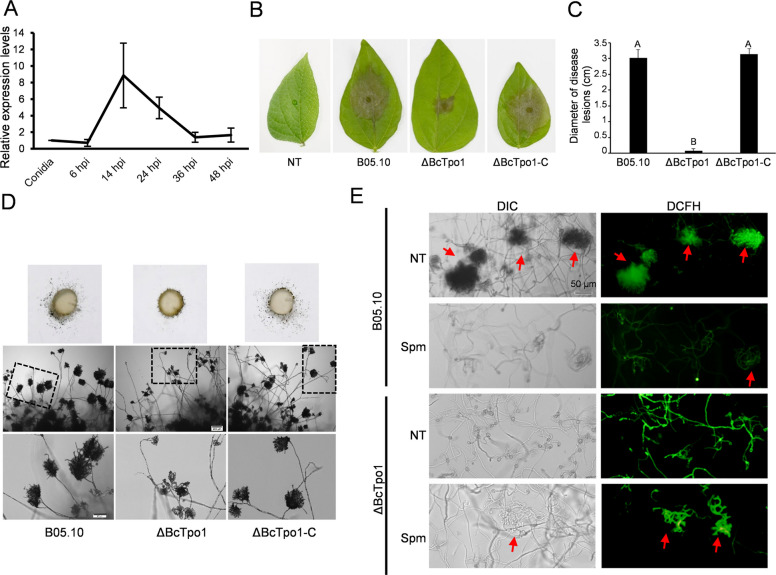
BcTpo1 is essential for virulence of *B. cinerea.* **A** Relative transcript levels of *BcTpo1* during *B. cinerea* infection. Samples were collected at the indicated time points post-inoculation (hpi). **B** Symptoms on mung bean leaves inoculated with conidia of B05.10, ΔBcTpo1 and ΔBcTpo1-C*.* The conidia suspension (10^5^ conidia/mL) was dropped on the mung bean leaves. **C** Lesion diameters on mung bean leaves from (B) scored at 3 DAI. The values on the bars followed by the same letters were not significantly different at *P* = 0.05. **D** Infection cushion development in *B. cinerea*. Mycelial plugs taken from the edge of the 2-day-old culture of the strains were placed on clean slides and incubated at 25 °C for 36 h. **E** ROS accumulation detected by DCFH-DA staining in infection cushions of B05.10 and ΔBcTpo1 with or without Spm treatment (12.5 μg/mL). Conidia were inoculated on onion epidermis and incubated for 36 h before examination. Infection cushions are indicated with red arrows

ROS production is a crucial response of plants to pathogen attack [25]. Since BcTpo1 is implicated in the sensitivity of *B. cinerea* to H_2_O_2_, it is likely associated with the pathogenicity of this fungus. To investigate its role in virulence, we first analyzed the expression levels of *BcTpo1* using qRT-PCR. As shown in Fig. 4A, *BcTpo1* was significantly up-regulated during infection, suggesting a correlation with virulence. Pathogenicity assays conducted on mung bean leaves revealed that ΔBcTpo1 exhibited lower pathogenicity compared to B05.10 and the complemented strain (ΔBcTpo1-C) (Fig. 4B, C). The impaired virulence of ΔBcTpo1 was further associated with defects in fungal invasion structures. ΔBcTpo1 produced fewer and smaller infection cushions compared to B05.10 (Fig. 4D). Additionally, ROS accumulation was predominantly observed in the infection cushions compared to B05.10 (Fig. 4E). In contrast, ΔBcTpo1 exhibited higher ROS levels within its hyphae. Notably, the addition of Spm reduced the ROS content in hyphae and promoted the formation of infection cushions in ΔBcTpo1 (Fig. 4E). Collectively, these results indicate that BcTpo1 is crucial for the virulence of *B. cinerea*.

## Discussion

BcTpo1 regulates fungal development and pathogenicity through a novel mechanism that modulates Spm-mediated redox homeostasis. The results indicate that (1) Spm specifically inhibits conidial germination, infection structure formation, and virulence in *B. cinerea*; (2) BcTpo1 is essential for hyphal growth, conidia/sclerotia formation, and melanin production; (3) ΔBcTpo1 significantly inhibits the formation of infection cushions, resulting in a marked reduction in pathogenicity of this fungus; (4) BcTpo1 maintains intracellular Spm homeostasis, its deletion leads to reduced Spm accumulation under oxidative stress; and (5) exogenous Spm can partially rescue the growth and virulence defects in ΔBcTpo1 by activating antioxidant enzymes such as SOD and POD, thereby scavenging excess ROS. In summary, our experimental findings demonstrate that BcTpo1 plays crucial roles in the growth, development, formation of infection structures, and pathogenic processes of *B. cinerea*.

PAs are essential cell constituents whose depletion results in growth inhibition [26]. Spm and Spd are both polyamines widely present in cells; however, they exhibit distinct differences in their structure, charge, metabolic regulation, and functional emphasis in biology. These differences collectively dictate their unique roles in cell proliferation, gene expression, oxidative stress resistance, autophagy induction, chromatin stability, and aging and disease processes [27, 28]. The differential effects of Spm and Spd observed in this study also indicated that their function in *B. cinerea*, Spm but not Spd**,** exhibits a significant impact on mycelial growth or conidial germination (Fig. 1 and S1). This difference may be attributed to one or more of the following factors: (1) distinct transport affinities, where BcTpo1 preferentially transports Spm over Spd; (2) differential recognition by downstream signaling components; or (3) the activation of distinct subsets of antioxidant enzymes or stress-responsive transcription factors by Spm and Spd.

Spm is essential for the process of cellular proliferation, such as normal growth, spore germination, and dimorphism in a variety of fungi [29]. In addition, Spm is the most crucial PAs in plant defense against pathogens during incompatible host–pathogen interactions [17]. The dual role of Spm observed in this study—exogenous high concentrations inhibit fungal growth, while endogenous Spm is required for oxidative stress tolerance—highlights a concentration-dependent hormetic effect. Similar biphasic responses have been reported for polyamines in other organisms. At low concentrations, Spm may act as a signaling molecule priming antioxidant defenses, whereas excessive Spm could generate oxidative stress through polyamine catabolism [18]. This concentration-dependent balance likely fine-tunes the fungal response to host-generated ROS during infection. Notably, upon pathogen infection, Spm is synthesized at the infection site as a host defense mechanism [16, 17, 30], and evidence suggests that plant PAs are targeted by numerous pathogens [31]. Thus, Spm serves as a critical node in the plant‒pathogen arms race. Our observations indicate that a high concentration of Spm also inhibited the growth, development, and pathogenic processes of *B. cinerea* (Fig. 1), thereby further confirming its crucial role in the virulence of the fungus.

PAs inhibit metal autoxidation, thereby suppressing the production of ROS [32]. An alternative indirect mechanism involves the transcriptional regulation of antioxidant genes [33, 34] or the direct activation of antioxidant enzymes [35, 36]. However, polyamine catabolism also produced H_2_O_2_, indicating a complex relationship between polyamines and ROS. In our study, the mechanism by which exogenous Spm increases SOD and POD activities in ΔBcTpo1 likely involves both direct and indirect effects. The observation that exogenous Spm increases SOD and POD activities in ΔBcTpo1 (Fig. 3F-G) indicates a relatively direct effect. However, our qRT-PCR (Quantitative Real-Time Reverse Transcription Polymerase Chain Reaction) data reveal that in the wild-type strain, Spm treatment downregulates antioxidant enzyme-encoding gene expression, whereas in ΔBcTpo1, these genes, including their regulatory factors (BcAp1 and BcSkn7), are already upregulated under basal conditions and are further induced upon Spm addition (Fig. S2). This finding suggests that Spm may also act indirectly. Distinguishing between these possibilities will require in vitro assays using purified enzymes and time-resolved measurements of ROS dynamics following Spm addition.

Tpo1 is a member of the major facilitator superfamily, which is involved in the detoxification of excess PAs in yeast [21, 22]. However, the polyamine excretion by Tpo1 is notably low in yeast [21]. Our data reveal a different mechanism in *B. cinerea*. The finding that ΔBcTpo1 accumulates less Spm under oxidative stress (Fig. 3E) suggests that BcTpo1 may mediate Spm uptake or retention under stress conditions, rather than efflux. This functional divergence could reflect species-specific adaptations to oxidative stress. Alternatively, other transporters (e.g., Tpo2-Tpo4 homologs) might compensate for efflux in the mutant, while BcTpo1 plays a specialized role in stress-responsive Spm homeostasis. Future studies using radioactive Spm uptake/efflux assays in heterologous systems would help clarify the precise transport direction of BcTpo1.

Beyond its role in Spm homeostasis, BcTpo1 is involved in diverse biological functions. In yeast, Tpo1 confers resistance to various toxic chemicals [21]. In this study, ΔBcTpo1 exhibited a wide range of developmental defects, including impaired hyphal growth, conidia/sclerotia formation, and melanin production, confirming that BcTpo1 serves as a core regulatory protein essential for normal cellular processes in *B. cinerea*. The increased melanin production in ΔBcTpo1 correlates with the upregulation of melanin biosynthesis genes (Fig. 2I). However, it remains unclear whether this is a direct consequence of BcTpo1 deletion or an indirect response to elevated oxidative stress. Melanin is known to protect fungi from various stresses, including oxidative stress [37, 38]. Thus, the increased melanin in ΔBcTpo1 may represent an adaptive response to compensate for impaired ROS homeostasis. Supporting this, the mutant exhibits elevated ROS levels (Fig. 3C-D), which could serve as a signal to upregulate melanin synthesis. Future studies using antioxidants to reduce ROS levels in ΔBcTpo1 would help determine whether melanin upregulation is a direct or indirect effect.

In this research, we report the regulatory mechanism that BcTpo1 governs development and virulence in *B. cinerea*. Spm inhibits the modulation of several key pathogenic processes, including conidial germination and the formation of infection structures. BcTpo1 is responsible for the efflux mechanism that regulates intracellular Spm concetrations. Its deletion results in less Spm accumulation specifically under oxidative stress conditions. Consequently, the mutant exhibits significantly impaired hyphal growth, infection cushion formation and pathogenicity. The introduction of exogenous Spm mitigates these deficiencies by activating antioxidant enzymes. Our findings reveal the critical role of BcTpo1 in polyamine metabolism, ROS balance, hyphal growth, and virulence, which may be harnessed for innovative antifungal strategies.

## Materials and motheds

### Strains and culture conditions

*B. cinerea* B05.10 Pers. Fr. [*B. fuckeliana* (de Bary) Whetzel] is commonly acknowledged as a benchmark reference strain [39]. The fungus was grown on Potato Dextrose Agar (PDA) plates, comprising 200 g of potato, 20 g of glucose, 20 g of agar, and 1 L of water.

### Growth tests

Mycelial plugs were freshly taken from the edge of a 3-day-old B05.10 colony and transferred onto PDA plates supplemented with Spm and H_2_O_2_, as indicated in the figure. These cultures were incubated at 25 °C for three days prior to examination. The relative inhibition of mycelial growth was quantified as [(C – T)/C] × 100, where T represents the diameter of the treated strain and C denotes the diameter of the control strain without treatment. Conidial germination rates were assessed on a hydrophobic surface (glass) in 1/4 PDB (Potato Dextrose Broth) after incubation for five hours at 25°. An untreated sample served as the control, and each experimental group was analyzed in triplicate. The percentage of conidial germination inhibition was evaluated using the formula RGI% = [(C – N)/C] × 100, where C refers to the conidial germination rate of the control, while N indicates that of the treatment.

### Pathogenicity test

The pathogenicity test was conducted on leaves of mung beans. Conidia of *B. cinerea* were collected from culture plates that had been grown for 10 days using a 10 mM glucose solution and then adjusted to a final concentration of 10^5^ conidia/mL. A 20 µl aliquot of this conidial suspension was applied to ten healthy, 3-week-old mung bean leaves that were unwounded, while a 20 µl solution of 10 mM glucose served as the negative control. After allowing for 3 days of incubation at 25 °C under 16 h of light, photographs of the tomato leaves were taken, and the diameters of the lesions caused by the disease were measured. Additionally, an assay to observe infection-related morphogenesis was carried out on onion epidermis, following previously established methods [40, 41].

### Construction of gene deletion and complementation strains

Gene deletion vectors were constructed employing a double-joint PCR (Polymerase Chain Reaction) method for *BcTpo1* [42]. The 5’ and 3’ flanking regions of *BcTpo1* and the hygromycin resistance gene cassette (HPH) were amplified using the primer pairs provided in Table S1. The resulting PCR products were transformed into B05.10 via protoplast formation and transformation techniques applicable to *B. cinerea* [43]. For the construction of the complementation vector, the plasmid pBS-neo served as the basis for this investigation [44]. The complete *BcTpo1*, including its promoter and terminator regions, was amplified from the genomic Deoxyribonucleic acid (DNA) of B05.10 and inserted between the NotI and SacI restriction sites of pBS-neo to construct the complementation plasmid. The primers used in this study are listed in the Supporting Information, Table S1.

### RNA extraction and quantitative reverse transcription PCR (qRT-PCR)

The expression levels of *BcTpo1* were tested by qRT-PCR using the 2^−ΔΔCt^ method [45]. For H₂O₂ and Spm treatments, fresh mycelium was collected from 3-day-old liquid cultures and resuspended in fresh PDB medium containing the indicated concentrations of H₂O₂ (5 mM) or Spm (6.25 μg/mL). The suspensions were incubated at 25 °C with shaking at 180 rpm for 3 h. After treatment, mycelia were immediately collected by filtration, washed twice with sterile water, and processed for further analysis. Conidial suspensions (10^6^ conidia/mL) were applied onto mung bean leaves. Following a 2-day incubation period, both conidia and mycelia from the strains, along with the plant tissue, were collected at intervals of 0, 6, 14, 24, 36, and 48 h. RibonucleicAcid (RNA) extraction, reverse transcription, and qRT-PCR were conducted according to a previously described protocol [44]. The actin gene was utilized as a reference for amplification. Three biological replicates were used for each sample.

### Spm analysis

Fresh mycelium of B05.10 and the mutants were treated with 10 mM H_2_O_2_ or Spm for 3 h prior to collection, while untreated samples served as controls. Spm was extracted with 5% perchloric acid (PCA) and analyzed using HPLC as described previously [46, 47].

### SOD and POD activity measurement

SOD (EC 1.15.1.1) and POD (EC1.11.1.7) were analyzed using the protocol described in (Superoxide Dismutase Activity Assay Kit, BC0170; Peroxidase Activity Assay Kit, BC0090, Beijing Solarbio Science & Technology Co., Ltd.). The mycelium treated with/without chemicals was processed and analyzed according to the manufacturer’s protocols.

For enzyme activity assays, mycelia (0.1 g fresh weight) were ground in liquid nitrogen and homogenized in 1 mL of extraction buffer. The homogenate was centrifuged at 8,000 × g for 10 min at 4 °C, and the supernatant was used for enzyme assays. Protein concentration was determined by the Bradford method using a BCA Protein Assay Kit (PC0020, Beijing Solarbio Science & Technology Co., Ltd.). SOD activity was measured at 560 nm, with one unit (U) defined as the amount of enzyme causing 50% inhibition of nitroblue tetrazolium photoreduction. POD activity was measured at 470 nm, with one unit defined as the amount of enzyme catalyzing 1 μg of substrate per minute per mg protein. All assays were performed in triplicate.

### Fluorescence microscopy

The conidia of *B. cinerea* were diluted in YEPD medium to a final concentration of 10^5^ conidia/mL. Following a 24-h incubation period at 25 °C with shaking at 180 rpm, the samples were supplemented with Spm and incubated for an additional 3 h at 25 °C prior to collection. Intracellular ROS accumulation was assessed using DCHF-DA (S0035, Beyotime) staining at a concentration of 10 mM [48].

### Statistical analysis

All experiments were conducted with a minimum of three independent biological replicates. Statistical analyses were performed using GraphPad Prism. Comparisons between two groups were analyzed by Student's t-test. Multiple group comparisons were performed by one-way analysis of variance (ANOVA). Significance levels are indicated as follows: *p < 0.05, **p < 0.01, ***p < 0.001, ****p < 0.0001; ns, not significant.

## Supplementary Information

Supplementary Material 1: Fig. S1. Spd had no significant effect on mycelial growth and conidial germination of B. cinerea.

Supplementary Material 2: Fig. S2. Heatmap showing the expression profiles of redox-related genes in the B05.10 and ΔBcTpo1 strains in response to Spm treatment. Mycelia were treated with or without Spm (12.5 μg/mL) for 3 h. Relative transcript levels of the indicated genes were determined by qRT-PCR. Data represent the mean of three independent biological replicates.

Supplementary Material 3: Table S1. Gene description and primers used in the study.

## Data Availability

The data that support the findings of this study are available from the corresponding author upon reasonable request.

## Abbreviations

*B. cinerea*: *Botrytis cinerea*
CCP: Cytochrome c Peroxidase
DCFH-DA: 2,7-Dichlorodihydrofluorescein diacetate
DNA: Deoxyribonucleic acid
MFS: Major facilitator superfamily
PA: Polyamine
PCA: Perchloric acid
PCR: Polymerase Chain Reaction
qRT-PCR: Quantitative Real-Time Reverse Transcription Polymerase Chain Reaction
PDA: Potato Dextrose Agar
PDB: Potato Dextrose Broth
POD: Peroxidase
PRD: Peroxiredoxin
Put: Putrescine
ROS: Reactive oxygen species
RNA: RibonucleicAcid
Spm: Spermine
Spd: Spermidine
SOD: Superoxide dismutase
