# Supplementary figures and images for: The MFS transporter BcTpo1 governs the oxidative stress response and infection of Botrytis cinerea
Source: Crop Health. 2026 Apr 22;4(1):12. doi: 10.1007/s44297-026-00074-7 (PMC13103187; doi:10.1007/s44297-026-00074-7)

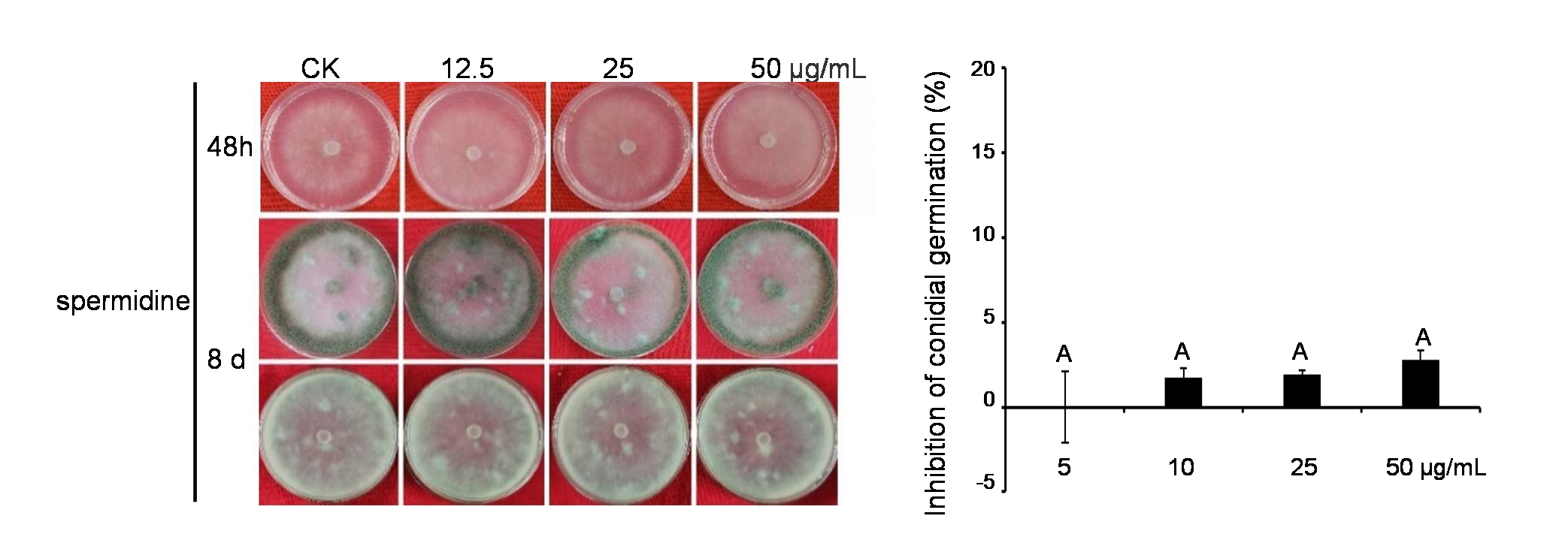

Supplement: Supplementary file 1 — Supplementary Material 1: Fig. S1. Spd had no significant effect on mycelial growth and conidial germination of B. cinerea. [file 44297_2026_74_MOESM1_ESM.tif]

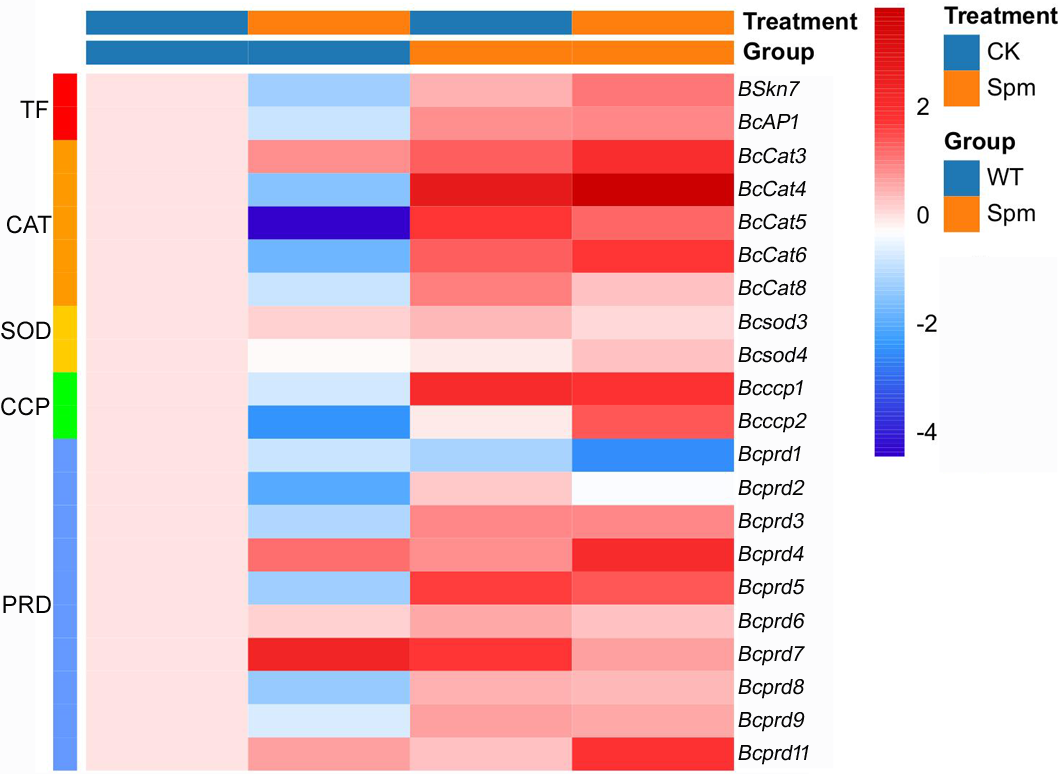

Supplement: Supplementary file 2 — Supplementary Material 2: Fig. S2. Heatmap showing the expression profiles of redox-related genes in the B05.10 and ΔBcTpo1 strains in response to Spm treatment. Mycelia were treated with or without Spm (12.5 μg/mL) for 3 h. Relative transcript levels of the indicated genes were determined by qRT-PCR. Data represent the mean of three independent biological replicates. [file 44297_2026_74_MOESM2_ESM.tif]
