# Supplementary material for: The MFS transporter BcTpo1 governs the oxidative stress response and infection of Botrytis cinerea
Source: Crop Health. 2026 Apr 22;4(1):12. doi: 10.1007/s44297-026-00074-7 (PMC13103187; doi:10.1007/s44297-026-00074-7)
Supplement: Supplementary file 3 — Supplementary Material 3: Table S1. Gene description and primers used in the study. [file 44297_2026_74_MOESM3_ESM.docx]

**Table S1. Primers used in the study.**

| Primer | Sequence (5'-3') | Relevant characteristics |
| --- | --- | --- |
| BcTpo1-up-F | TAGTGAGCCATGGTTGTTAT | Amplify *BcTpo1* upstream fragment for the construction of *BcTpo1* deletion mutants |
| BcTpo1-up-R | GACCTCCACTAGCTCCAGCCAAGCC CATGACTTCGCATTGGCAGG |  |
| BcTpo1-down-F | ATAGAGTAGATGCCGACCGCGGGTT TATGTTGGATGGGATGAC | Amplify *BcTpo1* downstream fragment for the construction of *BcTpo1* deletion mutants |
| BcTpo1-down-R | AGGGTCGCTACAAATAAG |  |
| HPH-F | GGCTTGGCTGGAGCTAGTGGAGGTC | Amplify the hygromycin resistant gene *HPH* |
| HPH-R | AACCCGCGGTCGGCATCTACTCTAT |  |
| BcTpo1-K-F | ATTACCAGTGCCTCATTCCG | Amplify the *BcTpo1* deletion cassette: up-HPH-down |
| BcTpo1-K-R | AAATCGAGGCAGACTAAC |  |
| BcTpo1-out-F | ATAGAGGATTGCCACCCCAC | Identification of *BcTpo1* deletion transformants |
| BcTpo1-out-R | CGCCATCAAAATGCAACG |  |
| BcTpo1-C-F | GCATTGATGTGTTGACCTCA TAGTGAGCCATGGTTGTTAT | Amplify full *BcTpo1* with including its own promoter fragment for complement of the *BcTpo1* deletion mutant |
| BcTpo1-C-R | TACTTACCTCACCCTTGGAAACCAT CACCACCGGTTCTGGCTTC |  |
| β-tubulin-F | TTCGACCCCAAGAACATGAT | *β-tubulin* for qPCR |
| β-tubulin-R | TAAGTCCTCGGGGAGGGAT |  |
| BcTpo1-RT-F | TGTGGTTTATTCTGGTTTGC | *BcTpo1* for qPCR |
| BcTpo1-RT-R | AAAATGCGATAGCCAAGAAG |  |
| BcPKS1-RT-F | TCAGCCAACGGGTCCTTATA | *BcPKS1* for qPCR |
| BcPKS1-RT-R | CTTGGGATCTATTGCTTCTGG |  |
| BcTHR-RT-F | TCTGGTTCTAAGGGTGCCATT | *BcTHR* for qPCR |
| BcTHR-RT-R | CCTTTCCGTTAACCCATTCA |  |
| BcMPS1-RT-F | ATTAGGGGACAACGACACATT | *BcMPS1* for qPCR |
| BcMPS1-RT-R | AATAGCCTTTAGGCCAGGAA |  |
| BcCMR1-RT-F | TCACGCATTGACACCTACACA | *BcCMR1* for qPCR |
| BcCMR1-RT-R | TTCCATGTTCCAATTCCTCG |  |
| BSkn7-RT-F | CACCCTCCATTGGGTTTAAA | *BSkn7* for qPCR |
| BSkn7-RT-R | TGGGTTGGGGTCACAGAATAT |  |
| BcAP1-RT-F | ACCCTTTTGCTCTTGATGAT | *BcAP1* for qPCR |
| BcAP1-RT-R | CATCAAGGTCAAATTCGCCT |  |
| BcCat3-RT-F | ACCAATAAAGAGTTCCAGTG | *BcCat3* for qPCR |
| BcCat3-RT-R | GTCACAACACCATACGAATC |  |
| BcCat4-RT-F | TGCCGTTACTTTTCTCTCCG | *BcCat4* for qPCR |
| BcCat4-RT-R | TCATCCTTCGCCATACCATT |  |
| BcCat5-RT-F | CGCCAACCCCAACTACCCAT | *BcCat5* for qPCR |
| BcCat5-RT-R | TTGCGAGTGTCCTCCTTAGC |  |
| BcCat6-RT-F | CAGCAACTCCAATCCAACGC | *BcCat6* for qPCR |
| BcCat6-RT-R | GAACCTCTCTCCAAAAATAC |  |
| BcCat8-RT-F | CAGTGAATGGCTGAAAGAAT | *BcCat8* for qPCR |
| BcCat8-RT-R | TGATCCAAGAGGCTGAAAAC |  |
| BcSod3-RT-F | TATTTAGCGGGCTCACCATT | *BcSod3* for qPCR |
| BcSod3-RT-R | ACTTTCTTTCCTCCTACATC |  |
| BcSod4-RT-F | CAAGTAAGAATGGTGGTGGT | *BcSod4* for qPCR |
| BcSod4-RT-R | GCCTTTCTGTTTTGGTATTG |  |
| BcCcp1-RT-F | TCGTTGCTTTGAGTGGTGCT | *BcCcp1* for qPCR |
| BcCcp1-RT-R | GAGAGATTCATCATTTGCGT |  |
| BcCcp2-RT-F | TTACAGACTTTTGCTTTCCC | *BcCcp2* for qPCR |
| BcCcp2-RT-R | TTACCCTTGCTATCTCTTCG |  |
| BcPrd1-RT-F | AGTAACACCCGAAGAGAAAG | *BcPrd1* for qPCR |
| BcPrd1-RT-R | CGTGCTTCTGTAGGCTTTTT |  |
| BcPrd2-RT-F | TACTATCGCTGGTTTCCTTG | *Bc**Prd2* for qPCR |
| BcPrd2-RT-R | GCGAGATTCCCAATAGCACC |  |
| BcPrd3-RT-F | TTTATTAGCAGCAGTGTTGC | *BcPrd3* for qPCR |
| BcPrd3-RT-R | AATGGGTAAAGCTTGATGTG |  |
| BcPrd4-RT-F | CTTTTGGCGACGAATACGGT | *BcPrd4* for qPCR |
| BcPrd4-RT-R | TCGACAAGATGCCCAACGGT |  |
| BcPrd5-RT-F | CGCACTCGACCTCAACATCG | *BcPrd5* for qPCR |
| BcPrd5-RT-R | ACTCAACCCACTCAACGCTG |  |
| BcPrd6-RT-F | AATCTTTGCTGGGGTTTTCG | *BcPrd6* for qPCR |
| BcPrd6-RT-R | AGTTGCCTTCGTCCATAGCC |  |
| BcPrd7-RT-F | GCAAACTCTTCCGACAAATC | *BcPrd7* or qPCR |
| BcPrd7-RT-R | GCCAAGATACTGCGTCACAT |  |
| BcPrd8-RT-F | GCGAGCAAATACCCTCCAAT | *BcPrd8* for qPCR |
| BcPrd8-RT-R | AACAGAGGCGAAAGAGAATT |  |
| BcPrd9-RT-F | TTCTTCATTTTTCGGTGTCG | *BcPrd9* for qPCR |
| BcPrd9-RT-R | AGAGAACCAGGCGTAGCAAT |  |
| BcPrd11-RT-F | GAAATCATCACCAATAACGG | *BcPrd11* for qPCR |
| BcPrd11-RT-R | ATCTCCTCACTCACTTTCTC |  |
